# Augmented reality: 3D image-guided surgery

**DOI:** 10.1186/1470-7330-15-S1-O8

**Published:** 2015-10-02

**Authors:** Archie Hughes-Hallett, Philip Pratt, James Dilley, Justin Vale, Ara Darzi, Erik Mayer

**Affiliations:** 1 Department of Surgery and Cancer, Imperial College, London, W2 1NY, UK; 2 Hamlyn Centre for Robotic Surgery, Imperial College, London, W2 1NY, UK

## Background

Over the last three decades, surgical practice has undergone a significant change with a move towards minimally invasive surgery (MIS) as the standard of care [1]. Although this has brought with it significant benefits, problems have also been associated with the advent of MIS. Perhaps the most substantial limitation associated with MIS is the loss of haptic feedback; this deficit is at its most extreme in robot-assisted surgery, where at present such feedback is lost entirely [2].

The *image-enhanced operating environment* looks to mitigate for the loss of haptic feedback by providing the surgeon with visual cues to the subsurface anatomy. The use of intraoperative image guidance can be divided into that used for operative *planning*, to facilitate the rapid identification of critical anatomical structures, for example, and that used for task *execution*, an example of which is tumour resection [2]. These two steps have very different requirements, with the first needing a large amount of anatomical information to be displayed without the need to account for tissue deformation or accurate registration, while the second requires less information to be displayed, but with much greater spatial accuracy.

## Methods

The solution proposed herein, the *image-enhanced operating environment*, utilises two different imaging modalities and plays on their respective strengths to meet the differing needs of the two outlined steps of *planning* and *execution*. The platform has been built around the index procedure of robot-assisted partial nephrectomy, although its potential application extends well beyond this scope.

The first step of operative *planning* utilises 3D reconstructions of preoperative cross-sectional imaging manipulated via a tablet-based interface [3]. This information was displayed to the surgeon both on the tablet and within the *da Vinci* console using the stereo TilePro™ function (Intuitive Surgical, Sunnyvale, CA).

The second step of *execution* utilises optically registered intraoperative ultrasound. Using a live imaging modality mitigates for the problems of deformation often faced when trying to use preoperative imaging for high precision guidance. The ultrasound data is used to create freehand 3D reconstructions which are overlaid onto the operative view [4].

## Results

To date, over 60 cases have been undertaken using the tablet-based planning component of *the image enhanced operating environment*. Over the course of this series, a subjective benefit has been demonstrated through the analysis of prospectively-collected questionnaire results. In addition, the platform has demonstrated objective safety, with no detrimental effects observed on outcome parameters. The use of registered ultrasound has been demonstrated *in vivo*[5], with results of an *ex vivo* study demonstrating potential efficacy awaited.

## Conclusions

Replacing haptic feedback with visual cues to subsurface anatomy offers a number of potential direct and indirect benefits to the patient, including improved resection quality and a reduction in positive surgical margins. In addition to these direct benefits, the use of an *image-enhanced operating environment* could potentially influence case selection, where surgeons are prepared to take on cases with more challenging anatomy via a minimally invasive approach, because of the improved understanding they are given by the image guidance platform.
